# Appearance of Environment‐Linked Azole Resistance in the *Aspergillus fumigatus* Complex in New Zealand

**DOI:** 10.1111/myc.70104

**Published:** 2025-08-26

**Authors:** Arthur J. Morris, Wendy P. McKinney, Sally A. Roberts, Sasiharan Sithamparanathan, Zain Chaudhry, Matthew C. Fisher

**Affiliations:** ^1^ New Zealand Mycology Reference Laboratory LabPlus, Auckland City Hospital Auckland New Zealand; ^2^ Respiratory Medicine and New Zealand Lung Transplant Service Auckland City Hospital Auckland New Zealand; ^3^ Department of Infectious Disease Epidemiology Imperial School of Public Health London UK

**Keywords:** antifungal resistance, antifungal treatment, *Aspergillus fumigatus*, azoles, ECVs, genotype, non‐wild type, resistance mechanisms, wild‐type

## Abstract

**Background:**

Until 2020, azole resistance in *Aspergillus fumigatus* complex isolates in New Zealand was due to *cyp51A* hot spot mutations. This report details the appearance of environment‐linked tandem repeat (TR)‐related azole resistance genotypes since 2021.

**Methods:**

Isolates were tested by broth micro‐dilution. Clinical Laboratory Standards Institute criteria were used to define wild type (WT) and non‐wild type (non‐WT) isolates, which were identified by ß‐tubulin gene sequencing and had their *cyp51A* genotype for azole resistance determined. Whole genome sequencing (WGS) was applied to two patient pairs of sequential WT and non‐WT isolates.

**Results:**

From January 2021 to June 2024, 15 of 147 (10.2%) *A. fumigatus* complex isolates were resistant or non‐WT for one or more azole agents. Genotyping detected hot spot mutations in four and TR‐associated resistance in nine. No mutations were detected in two isolates. Four of the five TR_46_ mutations were TR_46_/Y121F/T289A. Three of the four TR_34_ mutations were different. WGS of the paired isolates showed that the non‐WT isolates were distinct. Azole‐containing fungicides are available for home use from garden centres. Patients with TR‐associated resistance did not have any obvious exposure to azole‐containing fungicides. There was no evidence for healthcare‐acquired transmission.

**Conclusions:**

*A. fumigatus sensu stricto* isolates with TR‐mutations linked to environmental resistance are now present in New Zealand. Those at risk of invasive *A. fumigatus* infection should receive advice to avoid high‐risk exposures. Reintroducing monitoring of azole‐containing fungicides is recommended.

## 1 Introduction

*Aspergillus* spp. are the most common cause of invasive mould infections globally [1, 2]. The *Aspergillus fumigatus* complex is the most frequently encountered group, comprising more than 60 species, including *A. fumigatus* itself (*A. fumigatus sensu stricto*), with varying antifungal susceptibility profiles [3, 4, 5, 6, 7].

Triazoles are the cornerstone of recommended treatments for invasive aspergillosis (IA) [8]. In *Aspergillus* spp., azoles target an essential enzyme in fungal ergosterol synthesis encoded by the *cyp51A* gene. Major mechanisms of azole resistance in *A. fumigatus* include mutations at hot spots in the *cyp51A* gene (target modification), alone or in combination with tandem repeats (TRs) in the gene's promoter region (overexpression), or the upregulation of efflux pumps [9, 10]. Hot spot mutations are described in patients who have received courses of azole treatment, whereas TR‐associated resistance has been strongly linked to the acquisition of environmental strains which have been exposed to fungicides containing azoles used in agriculture [9, 11, 12, 13, 14, 15, 16, 17, 18]. Other agricultural fungicides, such as benzimidazoles and quinone outside inhibitors (QoIs), may co‐select for additional resistance mechanisms in environmental *A. fumigatus* populations. Recent genomic and phenotypic data demonstrate that mutations conferring resistance to these fungicide classes, specifically benA F219Y and cytB G143A, co‐occur with TR‐associated pan‐azole resistance in both environmental and clinical isolates, indicating that they too have arisen as a consequence of selection by fungicides, resulting in multi‐mode‐of‐action phenotypes [18].

While, except for voriconazole and isavuconazole for *A. fumigatus sensu stricto* [19, 20], there are no Clinical Laboratory Standards Institute (CLSI) interpretive criteria for susceptibility or resistance for moulds, epidemiological cut‐off values (ECVs) have been established for some species/complex‐antifungal agent pairings. An ECV defines the upper minimal inhibitory concentration (MIC) limit of wild‐type (WT) isolates, without acquired resistance mechanisms, and non‐wild‐type (non‐WT) isolates likely to harbour acquired resistance mechanisms [21, 22]. We have recently reviewed the antifungal susceptibility results of mould isolates performed in Auckland from 2001 to 2019 [23], and reported the emergence of azole resistance in local *A. fumigatus* complex isolates due to hot spot mutations in *cyp51A* [24]. This report describes the more recent appearance of TR‐associated azole resistance in New Zealand. We additionally leverage whole‐genome sequence (WGS) data of two pairs of longitudinal clinical isolates to shed light on possible mechanisms underlying the acquisition of resistance.

## 2 Patients and Methods

### 2.1 Isolates

Auckland City Hospital is a 1200‐bed tertiary/quaternary referral hospital with services covering haematology, oncology and transplantations. It is the national referral centre for lung transplants. This is a retrospective review of antifungal testing performed between January 2021 and June 2024. Fungal cultures were performed from a wide range of patient groups and body sites. Isolates were tested at Auckland City Hospital, and the detailed methods are reported elsewhere [23].

### 2.2 Antifungal Susceptibility Testing

All isolates were tested by the broth colorimetric micro‐dilution method, Sensititre YeastOne (SYO) (TREK Diagnostic Systems, West Sussex, England) following the manufacturer instructions. Endpoint interpretations followed CLSI methods [19, 25]. The minimal inhibitory concentration (MIC) endpoint was defined as the lowest concentration producing complete inhibition of growth for amphotericin B (AMB), itraconazole (ITC), posaconazole (POS), voriconazole (VCZ) and isavuconazole (ISA) [25]. The minimal effective concentration (MEC) for the echinocandins (caspofungin, micafungin (MICA) and anidulafungin) was defined as the lowest concentration producing small, rounded, compact hyphal forms compared to the hyphal growth of the growth control [25]. SYO plates were incubated at 35°C and were read at 24 h for echinocandins and 48 h for other agents.

WT isolates were defined by CLSI ECVs for AMB (2 mg/L), ITC (1 mg/L) and VOR (2 mg/L) [22]. POS has no endorsed CLSI ECV published, so the CLSI derived ECV of 0.25 mg/L was used [26]. VOR susceptibility was defined as ≤ 0.5 mg/L [19] and ISA as ≤ 1 mg/L [20].

Isolates A66545‐A66548 represent pairs of longitudinal isolates from two patients. A66545 and A66546, from specimens cultured 2 months apart, refer to the preceding WT and subsequent non‐WT isolate from the patient with isolate 3, Table 1. A66547 and A66548, from specimens cultured 10 months apart, are the WT and non‐WT isolates from the patient with isolate 4, Table 1.

**TABLE 1:** Antifungal susceptibility results (mg/L) for antifungal agents for *Aspergillus fumigatus* complex isolates: January 2021–June 2024.

			AMB	ITC	POS	VCZ	ISA	MICA	
Criteria for wild‐type (WT) isolates (mg/L)^a^			2	≤ 1	≤ 0.25	≤ 1	≤ 1		
Interpretive criteria for voriconazole and isavuconazole; susceptible, intermediate, resistant^b^						*S* ≤ 0.5 *I* = 1 *R* ≥ 2	*S* ≤ 1 *I* = 2 *R* ≥ 4		
Identification^c^, (year of isolation)	Specimen type^d^	Clinical background							*cyp51A* gene Resistance genotype
*A. fumigatus sensu stricto* (2021)	BAL	Chronic pulmonary aspergillosis	2	**> 16**	**> 8**	0.25 [S]	—	< 0.008	G54W
2 *A. fumigatus sensu stricto* (2021)	Sputum	Cystic fibrosis	**4**	1	**0.5**	1 [I]	—	< 0.008	F46Y/M172V/E427K (G98G/C454C)
3 *A. fumigatus sensu stricto* (2021)	BAL	Lung transplant	2	1	**0.5**	**> 8 [R]**	**> 8**	< 0.008	TR_46_/Y121F/T289A/(S49S)
4 *A. fumigatus sensu stricto* (2022)	BAL	Lung transplant	2	1	**1**	**> 8 [R]**	**> 8**	< 0.008	TR_46_/Y121F/T289A
5 *A. fumigatus sensu stricto* (2022)	BAL	Lung transplant	2	**> 16**	**2**	**> 8 [R]**	**> 8**	< 0.008	TR_34_/L98H/M172I/G448S
6 *A. fumigatus sensu stricto* (2022)	BAL	Bronchiectasis	**4**	**> 16**	**2**	**8 [R]**	**8**	0.015	N248K
7 *A. fumigatus sensu stricto* (2023)	BAL	Lung transplant	2	1	**0.5**	**> 8 [R]**	**> 8**	< 0.008	TR_46_/Y121F/T289A
8 *A. fumigatus* *sensu stricto* (2023)	BAL	Lung transplant	2	1	**0.5**	**> 8 [R]**	**> 8**	< 0.008	TR_46_/Y121F/T289A
9 *A. neoellipticus* ^e^ (2023)	BAL	Lung transplant	2	0.25	0.12	**2 [R]**	1	< 0.008	F46Y/G89E/M172V/E427K/G448S. Also 69 base pair insertion between amino acids 67 and 68
10 *A. fumigatus sensu stricto* (2023)	Sputum	Cystic fibrosis	2	**> 16**	**1**	1 [I]	**8**	< 0.008	TR_34_/L98H/S297T/F495I
11 *A. fumigatus sensu stricto* (2023)	BAL	Lung transplant	2	**16**	**1**	**4 [R]**	**4**	< 0.008	TR_34_/L98H
12 *A. fumigatus sensu stricto* (2023)	Lung tissue	Pneumothorax	2	1	**0.5**	**2 [R]**	**2**	< 0.008	No mutation detected
13 *A. fumigatus sensu stricto* (2023)	Bone^f^	Chronic granulomatous disease (CGD)	2	**> 16**	**2**	**> 8 [R]**	**> 8**	< 0.008	TR_34_/L98H/M172I/G448S
14 *A. fumigatus sensu stricto* (2023)	BAL^f^	CGD	2	1	**0.5**	**> 8 [R]**	**> 8**	< 0.008	TR_46_/Y121F/T289A
15 *A. fumigatus sensu stricto* (2024)	BAL^g^	Bronchiectasis	2	**> 16**	**1**	**4 [R]**	**8**	< 0.008	No mutation detected

Abbreviations: AMB, amphotericin B; ISA, isavuconazole; ITC, itraconazole; MICA, micafungin; POS, posaconazole; VOR, voriconazole.

^a^ Clinical Laboratory Standards Institute (CLSI) criteria used for amphotericin B, itraconazole, and voriconazole [22]. Posaconazole criterion from CLSI‐based methods [26]. WT = wild‐type. Non‐WT minimum inhibitory concentrations (MICs) in bold.

^b^ For *A. fumigatus sensu stricto*, not other species in the *A. fumigatus* complex [19, 20]. Resistant MICs in bold.

^c^ Isolates identified by β‐tubulin gene sequencing.

^d^ BAL, bronchioalveolar lavage or wash specimen.

^e^ Member of *A. fumigatus* complex.

^f^ Same patient, isolated from different sites during one hospital admission.

^g^ Morphologically different azole‐susceptible isolate present in the same sample culture. On retesting, identical results were obtained.

Isolates A66545‐A66548 additionally underwent susceptibility testing for benomyl, a benzimidazole fungicide that arrests cell division, and azoxystrobin, a QoI acting on cell respiration (energy production).

Benzimidazole and QoI fungicide growth testing was performed using 5 μL spot‐inoculations from glycerol stocks onto Sabouraud Dextrose Agar (SDA). For benzimidazole testing, isolates were plated on Nunc 4‐well dishes containing 1 mL SDA per well supplemented with 4, 8 and 10 μg/mL of benomyl and a no‐drug control. QoI testing was performed on SDA supplemented with 10 μg/mL of azoxystrobin. These assays were intended to support genotypic findings at the *benA* and *cytB* loci and were not formal susceptibility tests. A control isolate (Af293) was included for growth comparison. Cultures were incubated at 45°C for 48 h to minimise environmental fungal contamination and reflect the thermotolerance of *A. fumigatus* .

### 2.3 Identification and Resistance Genotyping

All voriconazole resistant or non‐WT isolates were identified by β‐tubulin gene sequencing [3, 27], and had their azole resistance mechanism determined by *cyp51A* gene sequencing [28].

From Jan 2021 to June 2024, we recorded the number of fungal cultures performed, their culture results, the number of *Aspergillus* spp. isolates, the number having susceptibility testing performed, and their susceptibility results. We also recorded the number of bronchioalveolar lavage (BAL) or washings (BW) having fungal cultures.

### 2.4 DNA Extraction and Whole Genome Sequencing (WGS)

DNA extraction followed standard methods as detailed in Supporting Information. Sequence alignment, bioinformatic and phylogenetic analysis were carried out as described by Rhodes et al. [15] Low‐confidence SNPs were filtered out if they met any of the following criteria: DP < 10 || MQ < 40.0 || QualByDepth < 2.0 || FisherStrand > 60.0 || MQRankSum < −12.5 || ReadPosRankSum < −8.0 || SOR > 4.0. Annotated sequences were interrogated for non‐synonymous single nucleotide polymorphisms (nsSNPs) in the coding region of *cyp51A* (AFUA_4G06890), *benA* (AFUA_1G10910) and *cytB* (AfuMt00001) which are associated with triazole, benzimidazole, and QOI resistance, respectively. Twenty‐three environmental *A. fumigatus* isolate WGSs from the New Zealand kākāpō ( *Strigops habroptilus* ) were included in the phylogeny as controls. The kākāpō, New Zealand's native endangered species of flightless parrot, experienced an outbreak of *A. fumigatus* in 2019 [29]. The best‐fitting phylogeny with maximum likelihood was uploaded to the Microreact project [30] and can be accessed at https://microreact.org/project/3WRHvNo3XECXoftCVSqPDF‐kakapo.

### 2.5 Case Histories, Antifungal Exposure and Environmental Risk Factors

Patient histories were summarised from electronic medical records and environmental risk factors for TR‐related azole resistance were obtained from patients with a structured questionnaire; see Supporting Information. Clinical infection status was determined by the EORTC and ISHLT definitions [31, 32].

The occurrence of several isolates with TR‐related resistance in our lung transplant group raised the possibility of a healthcare‐related outbreak. We undertook a review to determine if there were any construction or renovation projects patients could have been exposed to as well as reviewing clinic and rehabilitation appointments to see if TR‐isolate positive patients were present at the same time.

The two other laboratories in the country performing mould anti‐fungal susceptibility testing, Christchurch and Hamilton in the South and North Island respectively, were contacted and asked how many *Aspergillus* spp. isolates had been tested and their results for January 2021 to June 2024.

### 2.6 Ethics

The authors confirm that the ethical policies of the journal, as noted on the Journal's author guidelines, have been adhered to; the appropriate ethical review committee approval has been received. This study was approved by the National Health and Disability Ethics Committee, study number HDEC 2023 EXP18413.

## 3 Results

### 3.1 Fungal Cultures

Between January 2021 and June 2024, there were 5 511 routine clinical fungal cultures performed. From these, 343 *A. fumigatus* complex and 75 other *Aspergillus* spp. were isolated. Of these cultures, 2 006 (36%) were BAL/BW specimens, and from these, 181 *A. fumigatus* complex and 28 other *Aspergillus* spp. were isolated.

### 3.2 Susceptibility and Genotype Results

One hundred and eighty‐three *Aspergillus* isolates had susceptibility testing performed: *A. fumigatus* complex 147 (80%) (comprising 14 *A. fumigatus sensu stricto*, *A. neoellipticus* and *A. fischeri* one each, and 131 not further identified), *A. niger* complex 18 isolates, *A. flavus* complex nine and nine other species/complexes ( *A. nidulans* complex three, *A. terreus* complex two, *A. ustus* two and one unspeciated). Of the 183 isolates tested, 114 (62%) were from BAL/BW specimens.

The VCZ and ISA resistant and non‐WT isolates for azoles are summarised in Table 1. All 15 were from the *A. fumigatus* complex, 14 being *sensu stricto*. Four isolates had hot spot mutations, nine had TR‐mutations, and no resistant genotype was detected in two. Four of the five TR_46_ mutations were TR_46_/Y121F/T289A. Three of the four TR_34_ mutations were different; Table 1.

15 of 147 (10.2%) *A. fumigatus* complex isolates were resistant or non‐WT for one or more azole agents. The overall prevalence of voriconazole and isavuconazole resistance in *A. fumigatus* complex was 8.2% (12/147) and 8.7% (11/126) respectively. The prevalence of non‐WT for amphotericin was 1.4% (2/147), itraconazole 4.8% (7/147) and 9.5% for posaconazole (14/147).

None of the other species were non‐WT for the antifungals for which ECVs exist.

The two other laboratories tested 37 *Aspergillus* spp. isolates over the period; 30 were *A. fumigatus* complex. All were susceptible to voriconazole and WT for the other azole agents tested (Julie Creighton and Len Saunders, personal communication, January 2025). In total, for the 42‐month period, the prevalence of voriconazole resistance in the *A. fumigatus* complex in New Zealand was 6.8% (12/177).

Growth on SDA supplemented with benomyl and azoxystrobin was observed only for isolates A66546 and A66548, which were found to carry putative resistance‐associated mutations at *benA* and *cytB*, respectively (Table 2). This phenotypic profile is consistent with a potential functional effect of these mutations and supports their use as markers of environmental exposure in azole‐resistant strains.

**TABLE 2:** Characteristics of clinical and environmental (kākāpō) isolates included in the phylogenomic analysis (*n* = 27).

Isolate ID, number of isolates	Source	*cyp51A* allele	*benA* allele	*cytB* allele^a^
A66545	Clinical. Azole wild type isolate obtained before isolate 3 Table 1	K427E/E255D/T248N	WT	S146N/V119I
A66546	Clinical. Isolate 3, Table 1. Azole non‐wild type, isolated 2 months after A66545	TR_46_/Y121F/T289A/K427E/E255D	F219Y	G143A/V13I/R170S
A66547	Clinical. Azole wild type isolate obtained before isolate 4 Table 1	K427E/E255D/T248N	WT	WT
A66548	Clinical. Isolate 4, Table 1. Azole non‐wild type, isolated 10 months after A66547	TR_46_/Y121F/T289A/K427E/E255D/T248N	F219Y	G143A/V13I/R170S
1 isolate	Environmental	WT	WT	V13I
18 isolates	Environmental	TR_130_	WT	V13I
4 isolates	Environmental	TR_130_	WT	S146N/V119I

^a^ Mutations with no known link to azole resistance in *Aspergillus fumigatus* . S146N mutation for benzimidazole resistance and G143A for QoI resistance [18].

### 3.3 Clinical, Antifungal Exposure, Environmental Risk Factors and Infection Control

While patients with hot‐spot mutation isolates had different clinical backgrounds, most with TR‐isolates were lung transplant recipients (six of eight, 75%), Table 1. The median time post‐transplant to isolation of the resistant isolate was 18 months (range 5–100) and 4 months (range 2–13) between susceptible and resistant isolates. Azole treatment was common before the isolation of the resistant/non‐WT strain for patients with either hot‐spot (three of four) or TR‐associated (six of eight) mutations. Most patients, 12 of 14 (86%), did not meet the definitions for proven or probable infection [31, 32]. One patient had proven invasive aspergillosis (positive bone biopsy, isolate 13 Table 1) and another had probable infection (isolate 3 Table 1).

Those with TR‐associated resistance lived in both islands of the country and none lived in the same city. While several were frequent gardeners, none used azole‐containing fungicides or had known exposure to places where they were used. One was also involved with horticulture but used non‐azole‐containing fungicides on crops. Mulching material was used in one garden, but commercial compost products were not.

**FIGURE 1:**
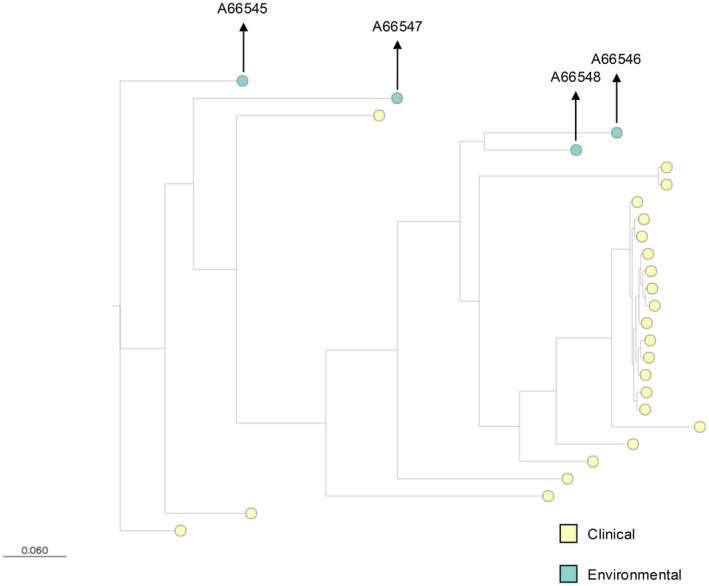
Maximum likelihood phylogeny of all study isolates (*n* = 27). Leaf tips are coloured by isolate origin, and the four clinical isolates are labelled accordingly. Environmental samples are *A. fumigatus* isolated from *kākāpō* ( *Strigops habroptilus* ). Wild‐type (A66545, A66547) and non‐wild‐type (A66546, A66548) represent sequential isolate pairs from two patients who acquired *cyp51A* TR‐associated azole resistance. Paired patient isolates A66545 and A66546 were separated by 60,805 SNPs, and A66547 and A66548 by 66,472 SNPs. The two non‐WT isolates, A66546 and A66548, were separated by 34,172 SNPs. In contrast, isolates from the *kākāpō* outbreak cluster were separated by a mean of ~2908 SNPs, consistent with a clonal outbreak. The phylogeny is available at: https://micrOreact.org/project/3WRHvNo3XECXoftCVSqPDF‐kakapo.

The four TR_46_‐positive isolates, all from lung transplant patients, had the same mutation, raising the possibility of common exposure. WGS of two of these four TR_46_ isolates, isolates 3 and 4 Table 1, showed them to be distinct strains and also distinct from their preceding WT isolates, Figure 1. There were no known construction or renovation projects in places transplant patients would have visited. Examination of clinic dates only found one date when Case 3 (with a resistant isolate then) and Case 7 were seen on the same day in late 2021. Case 7 then had five culture‐negative BW cultures and three with a susceptible *A. fumigatus* isolate before the TR_46_ isolate in early 2023, 17 months later. Cases 7 and 8, who had their TR_46_ isolates cultured a week apart in early 2023, had no identified common clinic dates and lived in different regions of the country.

There are approximately 160 active lung transplant patients in New Zealand and six (~4%) had isolates with TR‐associated resistance.

### 3.4 Genomic and Phylogenomic Analysis of WGS Data

Table 2 details the alleles found at the *cyp51A*, *benA* and *cytB* loci for environmental and patient isolates A66545–A66548. At the *benA* locus, non‐WT isolates A66546 and A66548 (which grew on benomyl agar) had the F219Y non‐synonymous single nucleotide polymorphisms (nsSNPs) whereas A66545 and A66547 were WT. At the *cytB* locus, A66546 and A66548 had the G143A nsSNP, whereas A66547 was WT and A66545 had S146N and V119I nsSNPs. All but one kākāpō environmental isolate carried a TR_130_ promoter repeat at *cyp51A*. While this structural variant has been recently described in environmental isolates from the UK, its functional significance remains unknown and it is not associated with azole resistance [33]. Similarly, the other loci mutations listed in Table 2 are not linked to azole resistance in *A. fumigatus* .

Figure 1 depicts the maximum likelihood phylogeny. Paired patient isolates A66545 and A66546 were separated by 60,805 SNPs; A66547 and A66548 by 66,472 SNPs. The two non‐WT isolates, A66546 and A66548, were separated by 34,172 SNPs. In contrast, isolates from the *kākāpō* outbreak cluster were highly similar, with a mean pairwise SNP distance of ~2908. The sequences of the patient isolates are avaialable in the NCBI database (https://www.ncbi.nlm.nih.gov/bioproject/PRJNA1302411, accession number PRJNA1302411).

## 4 Discussion

Over 42 months we encountered 15 voriconazole‐resistant or non‐WT isolates. Most, 13 of 15 (87%) had identifiable *cyp51A* mutations. As previously encountered, a number were due to hot‐spot mutations and associated with previous courses of azole treatment [24]. Our main and concerning finding was the recognition of TR‐associated azole resistance, which is considered to be environmentally acquired [9, 11, 12, 13, 14, 15, 16, 17, 18]. The proportion of resistance due to TR and hot‐spot mutations varies significantly between countries [34]. In the Netherlands, for isolates with *cyp51A* mutations, 90% and 10% were due to TR and hot‐spot mutations respectively, and 35% of azole‐resistant isolates had neither type of mutation [35]. In a UK series, however, where most isolates were from patients with chronic pulmonary aspergillosis and previous azole treatment, hot‐spot mutations dominated (over 90%) and TR mutations were uncommon, < 5% [36]. In our nearest neighbour, Australia, the proportion of hot‐spot, TR, or no *cyp51A* mutations detected in 12 isolates was 50%, 25% and 25% respectively [37, 38]. In a recent Spanish study, the proportions in 45 resistant isolates were 22%, 56% and 22% [39].

The clinical impact of azole resistance has been unclear as many earlier studies with small patient numbers have not shown outcome differences. In a recent large multicentre retrospective cohort, infection with triazole‐resistant aspergillosis was associated with 21% and 25% higher mortality at 42 and 90 days, respectively [40]. None of our patients died but only two had proven or probable infection. The country's 6.8% prevalence of resistant and non‐WT isolates is below the 10% threshold for the level of resistance where initial azole monotherapy is replaced by combination therapy of an azole in combination with echinocandin or liposomal amphotericin B [41]. For our lung transplant patients, the prevalence of 4% is below that suggested for a change in initial treatment. None of our patients with TR‐associated resistance had a poor clinical outcome, but ongoing monitoring will be required to ensure best treatment options are made.

WGS data provided further insights into the mechanism by which triazole resistance occurred in two patients previously treated with courses of azoles. Initial isolates were wild type at the *cyp51A* locus and did not exhibit phenotypic resistance to benzimidazoles or QoIs. Isolates obtained from serial sampling (A66546 and A66548) exhibited the TR_46_/Y121F/T289A allele, known to be associated with voriconazole resistance [42]. These triazole‐resistant isolates also harboured the F219Y and G143A nsSNPs in *benA* and *cytB*, respectively. These are known to confer high‐level resistance to benzimidazole and QoI fungicides in *A. fumigatus* [18]. The presence of resistance mutations to two classes of antifungal used exclusively in agriculture alongside TR_46_ strongly suggests that A66546 and A66548 are of environmental origin.

Additionally, it is clear from the phylogeny that the intra‐individual isolate pairs obtained through serial sampling of two patients (A66545—A66546 and A66547—A66548) are not genetically related given they were separated by an average of 63,639 SNPs. Patients could be colonised with several different environmental strains of *A. fumigatus* with varying susceptibility profiles, or isolates tested at different time points may represent transient colonisation. Subsequent exposure to triazole therapy may select out the organisms with resistance mutations (e.g., TR_46_/Y121F/T289A). How common this occurs is unknown. We occasionally encounter cultures with morphologically distinct colonies, and when we do, we perform susceptibility testing on both; the results are usually identical. However, cultures do occasionally have a mixture of susceptible and resistant isolates (as we observed with isolate 15 in Table 1). Alternatively, and more probably, patients may clear initial colonisation/infection with a single susceptible isolate, then become subsequently colonised with a resistant environmental isolate.

Agents used in agriculture must be registered and certified for use in New Zealand [43]. The register has more than 50 products containing demethylation inhibitor (DMI) azole agents known to have cross resistance with azoles used in medicine, eg. tebuconazole and difenoconazole [16, 41]. Previously the use of different classes of fungicides in New Zealand was reported on and, even though they comprised only a small proportion of all fungicide use, di‐ and triazole fungicides were used in agriculture in New Zealand [44]. However, this limited information on DMI use is almost 20 years old. To the best of our knowledge there is no monitoring of DMI fungicide use currently in New Zealand. The recommendation to register a product is on the basis that if used in accordance with the conditions for use it is “not likely to cause unacceptable risks to (amongst others) public health” [43]. Given the recent recognition of environmentally generated azole resistance in *A. fumigatus* it is unknown if the potential impact on human health is being considered. DMI resistance is known to occur locally in plant pathogens, eg. black spot or scab (*Venturia inaequalis*) on apples [45]. Given the relevance of DMI resistance from human health and food supply perspectives we support the call for better surveillance on pesticide use in New Zealand as well as a One Health approach to recognise and manage the risks [41, 44, 46, 47]. The need for monitoring is underscored by the observation that exposure of *A. fumigatus* to the new agrochemical fungicide ipflufenoquin, which has a similar structure and mode of action to olorofim, results in selection of strains resistant to olorofim [48].

The occurrence of azole resistance in such a short period raises the possibility of a common source exposure. However, the antifungal susceptibility results and genotypes of the four hot spot and three TR_34_ isolates showed they were different; WGS showed that two of the TR_46_ isolates were distinct. No other epidemiological links could be made between patients, and we have not identified a possible common exposure for the four transplant patients having isolates with TR_46_ mutations.

No patient with a TR‐mutation isolate had known exposure to azole‐containing fungicides in their home or work. Visits to two large garden centres in Auckland by one of the authors found that both had several azole containing fungicides available “over the counter”. All contained the triazole myclobutanil at various concentrations. Some patients were gardeners, but commercial compost use was not reported. Given the biology of composting and the association between increased probability of isolating both azole susceptible and resistant *A. fumigatus* from soil containing compost as well as compost having higher numbers than other garden samples, patients should be advised to handle compost in ways to reduce exposure [33, 41]. Local public health advice to reduce the risk of *Legionella* infection is for compost to be opened in a well‐ventilated area [49]. This advice, especially for high‐risk patients, eg. those with lung transplants, is also applicable to reduce exposure to massive numbers of *Aspergillus* spores as well. In addition, even though general environmental air exposure to *A. fumigatus* is common, including those with TR‐genotypes [50], it would seem prudent to advise lung transplant patients to also avoid home garden use of azole‐containing fungicides to avoid generating environmental hotspots, literally in their own back yard.

Most isolates came from Auckland, a city with the country's largest population and concentration of tertiary/quaternary clinical services. All non‐susceptible isolates had their MICs/MECs confirmed by repeat testing, and the susceptibility profiles of the different *cyp51A* mutations are consistent with previous reports [51]. Molecular methods determined the identity of all the non‐WT isolates. The results of all isolates tested in the country are included. However, this report has limitations: we had no ability to comment on the environmental use of azole‐containing fungicides, and WGS was not performed on all TR_46_‐positive isolates. We used the SYO microdilution method for susceptibility testing; while this method has 100% overall concordance with voriconazole, other azoles and amphotericin agreement varies between 90% and 95% [52].

## 5 Conclusion

New Zealand can be added to the list of countries with TR‐associated azole‐resistant *A. fumigatus* complex isolates. The results provide a baseline for monitoring this serious emerging antifungal resistance trend in *A. fumigatus* in New Zealand. Those at risk of invasive *A. fumigatus* infection should receive advice to avoid known high‐risk exposures. Monitoring use of azole‐containing fungicides in agriculture should be reintroduced.

## Author Contributions

**Arthur J. Morris:** conceptualization, investigation, writing – original draft, methodology, writing – review and editing, formal analysis, data curation, supervision. **Wendy P. McKinney:** data curation, methodology, validation, writing – review and editing. **Sally A. Roberts:** writing – review and editing, formal analysis, methodology, writing – original draft. **Sasiharan Sithamparanathan:** investigation, writing – review and editing, data curation, methodology. **Zain Chaudhry:** investigation, methodology, validation, visualization, writing – review and editing. **Matthew C. Fisher:** methodology, validation, writing – review and editing, supervision.

## Ethics Statement

The authors confirm that the ethical policies of the journal, as noted on the Journal's author guidelines, have been adhered to and the appropriate ethical review committee approval has been received. This study was approved by the National Health and Disability Ethics Committee, study number HDEC 2023 EXP18413.

## Conflicts of Interest

Matthew fisher has declared his funding sources. The other authors declare no conflicts of interest.

## Supporting information


**Data S1:** myc70104‐sup‐0001‐Supinfo.docx.

## Data Availability

The data that support the findings of this study are unavailable due to privacy or ethical restrictions.
