# Supplementary material for: Appearance of Environment‐Linked Azole Resistance in the Aspergillus fumigatus Complex in New Zealand
Source: Mycoses. 2025 Aug 26;68(8):e70104. doi: 10.1111/myc.70104 (PMC12379841; doi:10.1111/myc.70104)
Supplement: Supplementary file 1 — Data S1: myc70104‐sup‐0001‐Supinfo.docx. [file MYC-68-e70104-s001.docx]

**Appearance of environment-linked azole resistance in the *Aspergillus fumigatus* complex in New Zealand**

Supplementary material.

**Supplementary information. DNA extraction protocol.**

Isolates A66545-8 underwent DNA extraction and WGS as per the following protocol. Isolates were subcultured at 37℃ for 3 days on SDA in a 25 cm2 Nunc EasYFlaskTM. 10 ml of 0.05% Tween 20 was added to the flask, which was gently shaken to create a conidial suspension. A 1.8 ml aliquot of conidial suspension was transferred to a 2 ml Eppendorf and spun at 5000 rpm for 10 minutes. The pellet was resuspended in 300 μL of Yeast Cell Lysis Solution from the MasterPureTM Yeast DNA Purification Kit and vortexed (1). This solution was transferred into 2 ml Eppendorf containing Thistle 1.0 mm zirconium oxide beads (2). Tubes were transferred to a Qiagen Bead Beater and homogenised over two sets of three 45 second cycles at 30 Hz. Tubes were spun at 14,000 rpm for 2 minutes. Lysate was transferred into a new 1.5 ml Eppendorf and 1 μL of RNAse A was added. Tubes were incubated at 65ºC for 15 minutes followed by 15 minutes on ice.

20 μL of proteinase K from the Qiagen DNeasy Blood & Tissue Kit was added to each tube (3). A 200 μL aliquot of Buffer AL was added, and tubes were vortexed. Samples were incubated at 56℃ for 10 minutes. 200 μL of 100% ethanol was added, and samples were vortexed. This mixture was transferred to a 2 ml collection tube and centrifuged at 8000 rpm for 1 minute. 500 μL of Buffer AW2 was added, and the mixture was centrifuged at 14,000 rpm for 3 minutes. Finally, 36 μL of elution buffer was added, samples were incubated for 5 minutes at 22℃ then centrifuged at 8000 rpm for 1 minute. This step was repeated twice to maximise DNA yield.

DNA extracts were cleaned using the Zymo Research Genomic DNA Clean and Concentrator Kit as per manufacturer protocol (4). DNA concentration was confirmed using a QubitTM fluorometer. Samples were diluted with an elution buffer to a final concentration of 20 ng/μL in 25 μL and sent for WGS. Genome libraries were constructed using Illumina TruSeq Nano kit at Earlham facility. WGS was performed on an Illumina HiSeq 2500 sequencer, which generated 150 bp paired-end reads in high-output mode.

1. https://shop.biosearchtech.com/nucleic-acid-sample-preparation/dna-rna-extraction-and-purification-kits/dna-and-rna-purification-kits-and-reagents/masterpure-yeast-dna-purification-kit/p/MPY80200
2. Zirconium oxide beads 1.0mm [Internet]. Thistle Scientific. Available from: https://www.thistlescientific.co.uk/product/zirconium-oxide-beads-1-0mm/
3. DNeasy blood & tissue kits [Internet]. Available from: https://www.qiagen.com/us/products/discovery-and-translational-research/dna-rna-purification/dna-purification/genomic-dna/dneasy-blood-and-tissue-kit/
4. Zymo Research. Genomic DNA Clean & Concentrator®-25 Clean and concentrate large-sized DNA from any enzymatic reaction or impure preparation [Internet]. Zymo Research. Available from: <https://files.zymoresearch.com/protocols/_d4064_d4065_genomic_dna_clean_concentrator-25.pdf>

**Supplementary information: Questions for those with azole-resistant strains of *Aspergillus fumigatus* (ARAF).**

**Questions:**

1. Did you have any travel, including overseas travel, between [date, of previous isolate] and when you were seen at XX Hospital on [date]?
2. Do you recall any particular activities in the places you visited?

1. Can you please describe your house in [place]. Is it in town or more rural?
2. Do you have a garden, do you regularly tend any garden?
3. If so, what do you grow in your garden?
4. Do you use any fungicide/herbicide preparation(s) in your garden? (names of products if known)

1. Outside your home have you had exposure to plants / crops / flowers / plant waste / compost / garden centres / hot houses / greenhouses / wood chips?
2. Outside of your home do you have any exposure or use of herbicides, including fungicides?
3. What is your occupation?
4. Do you have hobbies?
